# Exploring Infertility in Saudi Arabia: Qualitative Insights into IVF Treatment Services and Policy Recommendations

**DOI:** 10.12688/f1000research.168014.1

**Published:** 2025-10-17

**Authors:** Ameina Ahmed D. Alshallali, Deema Saad AL Shawan

**Affiliations:** 1 Public Health, Imam Abdulrahman Bin Faisal University, Dammam, Eastern Province, Saudi Arabia

**Keywords:** Barriers to infertility treatment, in-vitro fertilization, male factor infertility, female factor infertility, primary infertility, secondary infertility, infertility, IVF

## Abstract

**Background:**

Infertility has become a significant issue that requires attention as its prevalence has increased globally and nationally in Saudi Arabia over the past few decades. This study aimed to explore patients’ perceptions towards infertility and in vitro fertilization-related services. Additionally, this study examined the barriers that prevent couples from seeking infertility treatment and IVF in Khobar and Dammam, Saudi Arabia.

**Methods:**

This study employed a qualitative, hybrid thematic analysis approach. Fifteen participants were included in the study. Participants were divided into three categories: infertility patients, infertility caregivers, and administrative staff working in infertility clinics.

**Results:**

This study identified four main themes: patients’ experiences, infertility services in Eastern Province, and perceived barriers to receiving treatment. Participants reported that they believed infertility was on the rise, especially male infertility. The reported obstacles included financial burdens, lack of awareness, societal influences, and lengthy, complex treatment processes.

**Conclusions:**

This study highlights the significant barriers that couples encounter in accessing infertility treatment. These findings emphasize the urgent need for improved public health policies, such as utilizing case management to support patients and incorporating insurance coverage for infertility treatments, along with enhanced support services.

## Introduction

Infertility is increasingly recognized as a pressing public health concern affecting millions of individuals and couples worldwide. According to the World Health Organization (WHO), approximately 17.5% of adults experience infertility. ^1^ Fertility rates in the Middle East and North Africa have fallen since the 1960s, dropping from an average of seven births per woman to 2.6 in 2021. ^2^ Furthermore, fertility rates in Saudi Arabia have steadily decreased, from 7.6 births per woman in 1960 to 2.46 births per woman in 2020. This surge has led to the global adoption of in vitro fertilization (IVF), which has significantly increased over the last 40 years. Since the first successful IVF birth in 1978, over eight million children have been born using this method. ^3^

IVF treatment was introduced in the Middle East during the 1980s, starting in Saudi Arabia. Currently, more than 35 centers in the Kingdom of Saudi Arabia (KSA) conduct approximately 20,000 IVF treatment cycles annually, with the majority of these facilities operating within the private sector. ^4^ The number of centers offering these services varies significantly by region in the Kingdom. In the Eastern Province, there is only one government center and four private centers that provide IVF services. ^5^

Despite this remarkable growth, various economic, social, cultural, and structural barriers prevent many individuals struggling with infertility from accessing IVF treatment. ^3^ The existing literature on perceptions of these services and the factors affecting accessibility in Saudi Arabia is limited. Nevertheless, global studies have shed light on patients’ perceptions and factors influencing their experiences with IVF treatment. For instance, a study conducted on the perceptions of infertility patients from six European countries found that factors such as a lack of knowledge of success rates, the high cost of treatment, and religious beliefs were obstacles to receiving those services. ^6^ Additionally, studies have found other factors that limit the number of couples seeking infertility treatment, including societal, mental, and economic challenges. ^7^

The most notable societal influence is the stigma surrounding male infertility, which can lead to a refusal of medical treatment, even when a male factor issue is suspected. ^9^

The emotional aspects of infertility can prevent patients from pursuing treatment. Gibson and Myers emphasized the need for counselors to be aware of infertile women’s coping needs and specific interventions to help them cope effectively with infertility stress. ^10^

This study aimed to examine the perceptions of patients with infertility regarding IVF services in the Eastern Province of Saudi Arabia. Additionally, we sought to identify factors that influence the process of seeking and receiving IVF treatment. The findings of this study will provide valuable insights for decision makers, enabling them to enhance the quality and accessibility of these services while fostering more culturally sensitive care. Furthermore, the results will inform the development of policies aimed at enhancing IVF services, ensuring that they are more effective and responsive to patient needs.

## Methods

This study employed a descriptive phenomenological qualitative method, specifically using a hybrid thematic analysis approach that incorporates both deductive and inductive coding. Initially, open coding was performed to organize the textual data and assign codes. This was followed by axial coding, which established the relationships between codes to create subcategories. Finally, selective coding is applied to connect these subcategories, ultimately leading to the development of overarching themes. ^11, 12^

The researchers implemented a purposeful sampling technique to select relevant participants. The inclusion criteria specified that the target population consisted of couples seeking IVF treatment in the Eastern Province of Saudi Arabia. Participants were categorized into three groups: patients, medical caregivers, and administrative staff at IVF centers. Those residing outside the Eastern Province were excluded because regional differences in healthcare delivery, culture, and available resources could significantly impact the findings. Furthermore, data collection and analysis were performed concurrently until data saturation was achieved with a sample of 15 participants. Data saturation refers to the stage in qualitative research in which additional data collection no longer generates new insights or themes. ^13^

Data were collected through individual in-person interviews conducted in infertility clinics in Khobar and Dammam, Eastern Province, Saudi Arabia, except for one interview conducted by phone at the participant’s preference. Participants received consent forms that were read and signed before the interviews were conducted. As for the participants interviewed over the phone, verbal consent was obtained prior to the interview. The interviews ranged from 45 minutes to two hours.

To improve the accuracy and reliability of the data, this study used a triangulation method for data sources, aiming to foster a thorough understanding of the phenomena under investigation. Data triangulation involves the use of multiple participants to verify the information gathered, ensuring that the findings are not biased or limited to a single perspective. In this study, data were collected from three different groups of participants: patients with infertility, caregivers, and administrative staff working in infertility clinics. In addition, data were collected from five hospitals. Two of the hospitals were government-run, and three were private. ^12^

Finally, the interview recordings were transcribed, and analyzed using ATLAS.ti after obtaining a copyright license. The analysis involved applying the necessary codes and themes. The study spanned six months, beginning with data collection in November 2023.

## Results

Of the 15 participants, five were OBGYN and infertility specialists, seven were patients, and three were administrative staff. Among the seven patients, five were female and two were male, with ages ranging from 19 to 46 years, all of whom were located in Khobar, Eastern Province. Their occupations vary, and include students, nurses, and some who are unemployed. The five medical caregivers consisted of obstetricians, reproductive medicine consultants, and a senior infertility consultant distributed between Khobar and Dammam. Lastly, the three administrative staff members worked as infertility clinic coordinators and nursing consultants, with two based in Khobar and one in Dammam [ Table 1].

**Table 1.** Participant profile.

Participant reference number	Category	Occupation	Gender	Age	Interview setting	City
Patient 1	Patient	Industrial and manufacturing field	Male	39	Private clinic	Khobar, Eastern Province
Patient 2	Patient	Student	Female	19	Governmental hospital	Khobar, Eastern Province
Patient 3	Patient	Unemployed	Female	32	Governmental hospital	Khobar, Eastern Province
Patient 4	Patient	Unemployed	Female	43	Governmental hospital	Khobar, Eastern Province
Patient 5	Patient	Nurse	Female	35	Private clinic	Khobar, Eastern Province
Patient 6	Patient	Nurse	Female	24	Private clinic	Khobar, Eastern Province
Patient 7	Patient	Housing administrator in the water desalination corporation	Male	46	Telephone call- private clinic	Khobar, Eastern Province
Physician 1	Medical caregiver	Obstetrician	Female	Did not disclose	Governmental hospital	Khobar, Eastern Province
Physician 2	Medical caregiver	Consultant in reproductive medicine and IVF	Female	Did not disclose	Private clinic	Khobar, Eastern Province
Physician 3	Medical caregiver	Consultant in reproductive medicine and IVF	Female	Did not disclose	Governmental hospital	Dammam, Eastern Province
Physician 4	Medical caregiver	Senior consultant in infertility and holder of a master’s degree in ART (assisted reproductive technology)	Male	Did not disclose	Governmental hospital	Dammam, Eastern Province
Physician 5	Medical caregiver	Consultant for infertility treatment and assisted reproduction	Female	Did not disclose	Private clinic	Khobar, Eastern Province
Admin 1	Administrative staff	Infertility clinic coordinator	Female	Did not disclose	Governmental hospital	Dammam, Eastern Province
Admin 2	Administrative staff	Nursing consultant	Female	Did not disclose	Governmental hospital	Khobar, Eastern Province
Admin 3	Administrative staff	Nurse supervisor	Female	Did not disclose	Governmental hospital	Khobar, Eastern Province

### Theme 1: Participants’ perceptions of infertility in the Eastern Province

The first theme explored the perceptions of infertility among all three participant groups in Khobar and Dammam. Seven out of 15 participants, including health professionals and patients, expressed that they believe infertility is increasing in Saudi Arabia despite the absence of publicly available data. For example, Physician 3 stated, “Recently, there has been an increase in infertility, but I don’t have any figures.” These comments highlight the importance of having access to infertility statistics as they influence perceptions of infertility among both health professionals and patients.

The participants also believed that primary infertility was more common than secondary infertility. Additionally, they believed that male factor infertility was the most common reason for seeking treatment. This view was supported by physicians, who mentioned that they had seen more cases of male factor infertility. For instance, Physician 2 stated that “male factor is really one of the things which we are facing. I can tell you that in some reported literature, they’re reaching up to 60% worldwide due to the male factor … I am sure if we currently conduct a study, we may find about 50 to 60% related to male factors” [ Table 2].

**Table 2.** Participants’ perceptions of infertility in the Eastern Province, Saudi Arabia.

Theme	Quotes
Infertility on the rise ^7^ *	“Recently, there has been an increase in infertility, but I don’t have any figures.”
Primary infertility is higher than secondary ^7^ *	“Mostly primary is higher than the secondary.”
The most common reason due to male factor ^6^ *	“Male factor is really one of the things which we are facing. I can tell you that in some reported literature, they're reaching up to 60% worldwide due to the male factor … I'm sure if we currently conduct a study, we may find about 50 to 60% related to male factors.”

* Number of quotes is indicated in parentheses.

### Theme 2: Perception towards infertility services in the Eastern Province

The second theme explored participants’ perceptions of infertility services in both the governmental and private sectors in Eastern Province. Physicians shared their views on the types of treatments they provided to patients. On the other hand, administrators described how they scheduled infertility patients and the services they offered. The patients explained where they sought treatment, whether in government or private clinics, which services were available to them, and their opinions on those services. This theme was further divided into five sub-themes: limited government resources, lack of male factor treatment, long waiting lists, unnecessary private sector services, and lack of insurance coverage [ Table 3].

**Table 3.** Perception towards infertility services in the Eastern Province.

Theme	Quotes
Limited resources in government services ^17^ *	“We are an educational government hospital. We don’t have a specialized clinic. We have IVF doctors, but they aren’t doing IVF procedures in the hospital.”
Unavailable male factor treatment necessities in the government sector in Eastern Province ^5^ *	“We do not have an andrology expert. Until now, we have not had a physician who specializes in treating men's reproductive-related issues, which is one of the basics of the department, honestly.”
Long waiting lists ^6^ *	“Since 2019, we have had 400 cases so far and approximately a thousand patients on the waiting list.”
Unnecessary services in the private sector ^3^ *	“… We were asked to undergo genetic testing, and I believe that’s cost us a lot of time, and it might delay one of the cycles. We don't think this is necessary in our case, at least.”
Lack of insurance coverage ^15^ *	“Of course, it’s not covered at all. Nothing. And by requesting any investigation, just by putting the reason as infertility, even if it was for a baseline investigation, it’s declined. Investigation, diagnosis, and infertility baseline investigation. The patients will pay for it out of pocket.”

* Number of quotes is indicated in parentheses.

### Limited governmental resources

According to the participants, currently, only one government center in the Eastern Province offers infertility treatment and accepts referrals from the region. The study participants also reported that a government hospital in Khobar had infertility specialists, but they did not perform procedures such as IVF. The government hospital receives patients with infertility issues, orders tests, performs ultrasound, and provides consultations. However, patients are referred to a single government infertility clinic in Dammam and placed on a long waiting list, referred to the government infertility clinic in Riyadh, or continue with necessary procedures at a private infertility clinic. It is essential to note that the scarcity of government infertility services was perceived as a significant issue in all three categories. As Admin 3 mentioned, “we are an educational government hospital. We did d not have a specialized clinic. We have IVF doctors, but they are not performing IVF procedures in the hospital.” Moreover, Physician 2 stated, “… the government or the public centers are not sufficient to accommodate the number of patients.” Additionally, Patient 3 said, “I wish they had an in vitro fertilization procedure. All hospitals are supposed to offer it.”

### Lack of male factor treatment in the Eastern Province

The participants disclosed that they believed that the only governmental infertility center in the region lacked an andrologist. Therefore, in cases requiring such a specialist, patients must be referred to a different region for treatment. Additionally, the participants reported that certain procedures related to male factors were not available at the government center. According to Admin 1, “We do not have an andrology expert. Until now, we have not had a physician who specializes in honestly treating men’s reproductive-related issues, which is one of the basics of the department.”

### Long waiting list

According to the participants, the current waiting list at the governmental infertility clinic in the Eastern Province is approximately 1,000 patients. This issue was mentioned by several patients, such as Patient 3, who noted that “IVF appointments can sometimes be held one year after being arranged.” Moreover, Admin 1 disclosed that “Since 2019, we have had 400 cases so far and approximately a thousand patients on the waiting list.”

### Unnecessary services in the private sector

Participants mentioned that some private hospitals ordered what they saw as unnecessary services, such as genetic testing. According to Patient 1, “… We were asked to undergo genetic testing, and I believe that it cost us a lot of time, and it might delay one of the cycles. We do not think this is necessary in our case, at least.”

### Lack of insurance coverage

Physicians, patients, and administrators in this study emphasized the importance of including infertility treatments under insurance coverage, stating that it is a medical condition that individuals should be able to treat. Similar to other health issues, it requires consultation and care. Physician 1 said about having children, “It’s one of the basic needs in life. It is not an accessory or a supplementary need; on the contrary, having children is one of life’s necessities, especially in Arab culture.” This view was echoed by Patient 6, who said, “It is expensive, and I do not know why insurance does not cover it, honestly, it’s unfair. It is the same as any health condition; it should be treated similarly. How is the fault of the person if they cannot have children?” Additionally, Admin 2 mentioned, “We do not have this service in our educational governmental hospital and the people struggle to save for a cycle to do it in a private place and the insurance usually they do not cover the IVF cycle. It’s very stressful.”

### Theme 3: Perceived barriers facing infertility patients

The third theme concerns the perceived barriers that prevent patients with infertility from seeking treatment from the participants’ perspective. This theme was further divided into five sub-themes: financial burden, lack of awareness, societal influences, the duration and complexity of the process, and the impact of these factors on individuals [ Table 4].

**Table 4.** Perceived barriers facing infertility patients seeking IVF, in the Easter Province, Saudi Arabia.

Theme	Quotes
Financial burden ^20^ *	“Patients have a huge financial burden because most insurance companies do not cover it. Having a few public hospitals creates a lot of emotional stress and financial stress on patients.”
Lack of awareness and proper guidance ^13^ *	“I feel that I was late in seeking treatment due to unawareness. If I had known earlier on, I could’ve gotten treatment.”
Limited in fertility centres ^10^ *	“For us, as an educational governmental hospital, since it is only a clinic and there are no procedures, giving is limited only to the lab work and ultrasound, which means we can only do simple things that do not complete the entire process.”
Societal influences ^11^ *	“We have a blaming culture. We have this pressure from family and society. Come on, you didn’t get pregnant. Come on, bring a brother or sister.”
Duration and complexity of the process ^4^ *	“Travelling to do such a procedure is not an easy decision because it's not a one-day procedure. You know, it's a back-and-forth process, and it will take about a month for various ultrasounds, blood work, follow-ups, and sometimes the patient herself needs to be in complete rest. So, you need to rent accommodations. It's a hassle.”

* Number of quotes is indicated in parentheses.

### Financial burden

The most common obstacle mentioned by patients is the financial burden of the infertility treatment. For example, Patient 3 expressed her view on how costly infertility treatments can be by saying, “I might say that another person can go to a private clinic and pay twenty thousand or thirty thousand, but others cannot pay such an amount.” Physicians and administrative staff also confirmed this, sharing their opinions on the high costs of these procedures and their beliefs that they should be covered by health insurance. According to Physician 2, “… Patients face a huge financial burden because most insurance companies do not cover it. Having a few public hospitals creates a lot of emotional and financial stress on patients.” Admin 2 also shared her perspective on how cost impacts seeking treatment by saying, “… I think many no longer seek IVF treatments, although they are desperate for a child because they can’t afford it.”

### Lack of awareness

Infertile patients perceived a lack of awareness and proper guidance, as they sometimes felt lost and unsure how or when to take the next step, since they were not guided properly. Some patients reported that their lack of knowledge prevented them from seeking treatment because they were unaware of the consequences of not seeking treatment at an early stage. For instance, physician 3 stated that “others might have a lack of information; they may not know that IVF is available in government hospitals, so that might delay seeking treatment.” Another reason is wasting time by visiting non-specialised clinics, which also delays seeking proper management. This finding highlights the importance of public health interventions to promote infertility awareness.

### Societal influences

Another barrier mentioned was societal influences and the pressure to have children, which can have the opposite effect and discourage participants from seeking treatment because they do not want to admit to family members that they are suffering from infertility. Patient 6 said, “I felt pressured by family members to have children. I was asked why she had not had children yet, and I felt I would let my family down if they knew about her infertility struggles.” This also influenced her decision not to seek treatment, since she was afraid that it would be unsuccessful and disappoint those around her. She also stated that “we have a blaming culture … we have this pressure from family and society…”

### Duration and complexity of the process

Infertility treatment can vary greatly, but it is often a lengthy process. Patient 1 described the level of commitment required during IVF cycles, emphasizing the importance of careful planning due to the precision needed in daily injections, oocyte retrieval, and embryo transfer. According to Patient 1, “this process demands not only a high level of commitment but also emotional taxation, which can discourage patients from proceeding with treatment.” This issue is amplified by a lack of relevant services in the region, which prompts patients to travel frequently. According to Patient 3, traveling to perform such a procedure is not an easy decision because it is not a one-day procedure. You know, it is a back-and-forth process, and it will take about a month for various ultrasounds, blood work, follow-ups, and sometimes the patient needs to be in complete rest. Therefore, you need to rent accommodations. It’s a hassle. Physician 1 added, “Another burden is that some patients struggle to adhere to the treatment plan because it requires frequent visits. IVF typically involves at least four visits to the clinic for procedures and embryo transfers. However, many patients have work commitments and cannot take time off for every appointment, which complicates compliance.”

## Discussion

This study aimed to discover participants’ attitudes towards infertility and IVF-related services, and to explore the barriers to seeking treatment. Key findings highlight that infertility is perceived to be on the rise, with a significant emphasis on male factor infertility as a prevalent issue. Participants reported barriers to accessing infertility services, including financial burden, lack of awareness, societal influences, and lengthy, complex treatment processes. The limited availability of government resources and the absence of insurance coverage for infertility treatments further exacerbate these challenges.

One notable finding was that patients expressed that a major obstacle is the length and complexity of the IVF treatment process, especially when most couples go through more than one cycle before success. A study conducted in the UK on live birth rates associated with repeated IVF cycles supported this finding, finding that three out of 10 women succeeded with their first cycle of IVF, while seven out of 10 succeeded by their sixth cycle. ^14^

In this study, the financial burden was another commonly cited barrier. This result aligns with that of a qualitative research conducted by Mosalanejad et al., who stated that treatment cost is the greatest barrier, even for people with high incomes. ^8^ Similarly, Insogna et al. mentioned that cost was reported as a barrier to care by 62.8% of the respondents. ^15^

The patients also expressed that they felt that they were not properly guided or informed. This prevented them from seeking treatment, either because they lacked knowledge of their condition or when to take the next step, which could lead to discontinuation of treatment, which aligns with the findings of Mosalanejad et al. ^8^

Another factor shared by participants was that one governmental center offering infertility treatment in the Eastern Province was not sufficient. This obstacle was also supported by Fahimi et al., and Mosalanejad et al. also mentioned the lack of infertility centers as a barrier. ^8, 16^

Another obstacle reported by several participants was the stigma surrounding infertility and its societal influence. Patients shared experiencing pressure from their families to conceive and fear disappointing them if the treatment failed. Cultural stigma surrounding male infertility has also been noted. Traditionally, women were seen as responsible for infertility, and men were hesitant to get tested due to fear of judgment, stigma, or damage to their masculinity. Agarwal et al. also recognized this issue and suggested increasing awareness and encouraging society to reduce barriers to infertility stigmas rooted in cultural beliefs. ^17^

The lack of sufficient health insurance coverage does not include infertility treatment. Therefore, these procedures can be costly for patients, especially for IVF, which creates a barrier to treatment. The participants mentioned that insurance should cover infertility treatment because infertility is a medical condition similar to any other condition. This finding was supported by Jain et al. and Peipert et al., who concluded that comprehensive mandated insurance coverage increased the utilization of these services, but decreased multiple birth rates. ^18, 19^ This factor was also supported by Al-Turki, who noted that a limited number of government hospitals in the Eastern Province offered IVF. The demand for more government infertility centers is clearly high due to the lack of insurance coverage and the number of patients who cannot afford to pay out-of-pocket for treatment in the private sector. ^5^

One limitation of this study was the lack of current infertility statistics in Saudi Arabia. Owing to the sensitive nature of the research topic, it was challenging to find patients, particularly men, who were willing to share their experiences. As a result, most participants were women, which limits the male perspective in this study. Additionally, information about the private sector is not publicly available, and staff at private clinics are hesitant to be interviewed or share how they manage their infertility clinics and patients. This may have affected the study’s results by limiting insights into private clinic administrative perspectives and emphasizing those of government hospitals.

## Conclusions

This study examines public perceptions and attitudes toward IVF-related services to identify the barriers encountered when seeking treatment. These findings reveal a growing concern regarding infertility, particularly highlighting male factor infertility as a significant issue. Participants identified several obstacles to accessing infertility services, including financial constraints, insufficient awareness, societal pressures, and the lengthy and complicated nature of the treatment processes.

Additionally, the limited availability of government resources and lack of insurance coverage for infertility treatments further intensify these challenges. Patients without insurance coverage should be able to receive treatment at government clinics. Moreover, there should be sufficient government clinics to accommodate patients who cannot afford treatment and who lack insurance coverage. ^20^

Owing to the challenges encountered when interviewing administrative staff in private infertility clinics, there should be greater transparency of information, especially with researchers. This will help provide a comprehensive understanding of infertility services available in both the government and private sectors. Access to data can support further research on infertility and improve current infertility services by offering information and insights to decision makers. Dyer and Zegers-Hochschild highlighted the potential impact of registries on reproductive health. By utilizing registry data, individual centers and countries can benchmark their performance against national, regional, and global standards, thereby providing valuable insights for research. This aligns with the findings of Abduljabbar and Amin, who noted that Saudi Arabia could enhance its international contributions to Assisted Reproductive Technology practices by establishing a government-managed IVF registry, thereby facilitating the global exchange of information. ^21^

Finally, to address one of the patients’ main concerns, navigating the complex and lengthy process of infertility treatment, we propose the use of case management. This policy recommendation can lead to the development of a systematic method in which a specialized professional, typically a nurse or care coordinator, assists patients in navigating intricate IVF procedures. Furthermore, research has demonstrated that this approach can enhance patient satisfaction. It also has the potential to alleviate anxiety and depression in individuals who have experienced pregnancy loss following in vitro fertilization and embryo transfer. Implementing such a policy in Saudi hospitals could streamline the process and positively impact patients’ mental health and overall quality of life. ^22^

## Ethics and consent

Ethical approval in accordance with the Declaration of Helsinki was obtained from the Institutional Review Board of Imam Abdulrahman Bin Faisal University. The IRB number was IRB-PGS-2023-03-259. All participants’ information and interviews were handled with strict confidentiality. Before the interviews began, the participants signed consent forms to confirm their approval to participate.

## Data Availability

The dataset is not publicly available due to research data-sharing restriction policies at our institution. Data and interview transcripts can be made available after removing participant-identifiable information. To request access to the data, please email the author, Ameina Alshallali, at ameina.alshallali@gmail.com. Access will be granted on the condition that you provide the purpose for requesting the dataset.
