# Supplementary material for: Exploring Infertility in Saudi Arabia: Qualitative Insights into IVF Treatment Services and Policy Recommendations
Source: F1000Res. 2026 Mar 31;14:1130. Originally published 2025 Oct 17. [Version 3] doi: 10.12688/f1000research.168014.3 (PMC13320124; doi:10.12688/f1000research.168014.3)
Supplement: Peer review history

# Reviewer report: Reviewer response for version 3

**DOI:** 10.5256/f1000research.197540.r482012

**Published:** 2026-06-30

**Authors:** Lucy Khofi

**Affiliations:** 1 University of the Witwatersrand, Johannesburg, South Africa

Thank you for the opportunity to review this manuscript. The article addresses an important and underexplored public health issue. The topic is timely and relevant, particularly given the increasing recognition of infertility as a public health concern and the need for more accessible, culturally sensitive reproductive health services.

 However, the manuscript would benefit from further strengthening in several areas before indexing.

**Major comments**

 First, the manuscript remains largely descriptive and would benefit from a stronger analytical framing. The barriers are clearly listed, but the discussion could go further in explaining how financial, institutional, social, and gendered barriers interact to shape unequal access to infertility treatment. For example, lack of insurance coverage, limited governmental IVF services, long waiting lists, stigma, and poor guidance are not separate issues only; together, they produce structural exclusion from reproductive healthcare. The authors could strengthen the manuscript by framing infertility treatment as part of broader sexual and reproductive health access and reproductive justice.

 In this regard, the discussion would benefit from engagement with literature on structural barriers to sexual and reproductive health services, especially work showing how economic insecurity, stigma, gendered vulnerability, and health-system exclusion restrict reproductive healthcare access. Although not infertility-specific, the following article may be useful for strengthening the broader framing of reproductive healthcare access and structural barriers: **Reference 1**. The authors could use this type of literature to position IVF access within wider debates on inequality, reproductive autonomy, and access to care.

 Second, the methodological section requires more detail and transparency. The authors state that they used a descriptive phenomenological qualitative method and hybrid thematic analysis, but the analytical process needs to be described more clearly. The manuscript should explain how the interview guide was developed, how participants were recruited, whether interviews were conducted in Arabic or English, how transcription and translation were handled, how coding was conducted, whether more than one researcher coded the data, and how disagreements in coding were resolved. The authors should also explain how data saturation was assessed, especially given the small sample size and the inclusion of three different participant groups.

 Third, the authors should be cautious in making broad claims about male-factor infertility. This is an important finding, and it is valuable that participants raised it. However, the study included only two male patients, and therefore the male patient perspective is limited. Claims about male infertility, men’s treatment-seeking behaviour, and stigma should be clearly grounded in the data and presented cautiously. The authors may also want to distinguish between what was reported by physicians, administrators, and patients.

 Fourth, the results section would benefit from richer qualitative analysis. At present, some sections read as a summary of barriers rather than an interpretation of participants’ lived experiences. The authors could include more detailed quotes and provide deeper interpretation of what these quotes reveal about stigma, uncertainty, fear of treatment failure, pressure from family, and the emotional burden of infertility. This would strengthen the qualitative contribution of the paper.

 Fifth, the policy recommendations are important, especially regarding insurance coverage, public-sector IVF provision, a national registry, awareness campaigns, and case management. However, these recommendations should be more explicitly linked to the study findings. For example, the recommendation for case management should be connected directly to participants’ accounts of lack of guidance, the length and complexity of treatment, repeated visits, and the emotional stress of navigating services. Similarly, the recommendation for insurance coverage should be linked to the strong evidence in the results about financial burden and out-of-pocket costs.

**Minor comments**

 The manuscript would benefit from careful language editing. There are several awkward or unclear phrases, such as “limited in fertility centers,” and some sentences require grammatical correction. The ethics section also contains repeated wording: “Before the interviews began,” which should be corrected.

 The authors should ensure consistency in terminology. For example, they should use either “governmental” or “public-sector” consistently throughout the paper.

 The tables are useful, but the authors should ensure that the number of quotes and the themes are presented clearly. Some table headings and theme labels could be refined for clarity.

 The authors should avoid overgeneralising from the qualitative findings. Since this is a small qualitative study in two cities in the Eastern Province, the conclusions should be framed cautiously and presented as context-specific insights rather than general claims about infertility care across Saudi Arabia.

**Conclusion**

 This is a relevant and valuable qualitative study with clear public health and policy significance. The manuscript makes an important contribution by highlighting the barriers faced by infertility patients in accessing IVF services in the Eastern Province of Saudi Arabia. However, the paper would be stronger if the authors deepened the analysis, improved methodological transparency, linked policy recommendations more directly to the findings, and strengthened the discussion through broader sexual and reproductive health access literature.

**Recommendation:** Approved with reservations / minor to moderate revisions.

Is the work clearly and accurately presented and does it cite the current literature?

Yes

If applicable, is the statistical analysis and its interpretation appropriate?

Yes

Are all the source data underlying the results available to ensure full reproducibility?

Yes

Is the study design appropriate and is the work technically sound?

Yes

Are the conclusions drawn adequately supported by the results?

Yes

Are sufficient details of methods and analysis provided to allow replication by others?

Yes

Reviewer Expertise:

Public Health

I confirm that I have read this submission and believe that I have an appropriate level of expertise to confirm that it is of an acceptable scientific standard, however I have significant reservations, as outlined above.

## References

[1] : Food insecurity, intimate partner violence, and barriers to sexual and reproductive health care among women in Lorentzville, South Africa. *Social Science & Medicine* .2025;369: 10.1016/j.socscimed.2025.117785 10.1016/j.socscimed.2025.117785 39951869

# Reviewer report: Reviewer response for version 2

**DOI:** 10.5256/f1000research.196152.r460091

**Published:** 2026-03-02

**Authors:** Emmanuel Ifeanyi Obeagu

**Affiliations:** 1 Africa University, Mutare, Zimbabwe

The manuscript needs major revision in the areas pointed below:

**Major Revision Comments – Abstract**

1. **Insufficient Conceptual Precision in the Background**
  The background section is overly broad and descriptive, lacking a clear articulation of the specific knowledge gap this study addresses. While infertility prevalence is mentioned globally and nationally, the abstract does not sufficiently justify *why* patients’ perceptions and barriers in Khobar and Dammam warrant focused investigation, nor how this study advances existing literature. The authors should explicitly state the unmet research gap and the study’s unique contribution.
2. **Objectives Require Greater Clarity and Analytical Focus**
  The study aims are stated in a generalized manner and conflate perceptions, service evaluation, and barriers without clearly distinguishing primary and secondary objectives. The abstract should clearly specify whether the central focus is patient perception, service accessibility, systemic barriers, or policy implications, and how these components are analytically linked.
3. **Methodological Description Lacks Rigor and Transparency**
  The methods section is insufficiently detailed for a qualitative study. The term “qualitative, hybrid thematic analysis” is vague and not adequately explained. The abstract should briefly clarify what is meant by “hybrid” (e.g., inductive–deductive), the data collection method (e.g., interviews, focus groups), and the rationale for including three distinct participant groups. Additionally, justification for the small sample size (n=15) and its adequacy for thematic saturation is absent.
4. **Results Section Is Incomplete and Conceptually Inconsistent**
  There is an inconsistency in the reporting of themes: the abstract states that *four* themes were identified but lists only *three*. This is a major structural flaw. Furthermore, the results are presented at a superficial level, largely restating common assumptions (e.g., financial burden, societal influence) without highlighting novel, context-specific insights. The authors should succinctly articulate the most salient and distinctive findings emerging from the data.
5. **Conclusions Are Overgeneralized and Not Fully Grounded in the Results**
  The conclusions section introduces policy recommendations (e.g., case management, insurance coverage) that are not clearly tied to the reported findings within the abstract. The authors should ensure that conclusions logically flow from the stated results and avoid introducing new concepts not previously mentioned. The implications should be framed cautiously, reflecting the qualitative nature and limited sample size of the study.
6. **Language, Structure, and Technical Accuracy Require Improvement**
  The abstract contains grammatical errors (e.g., “his study” instead of “This study”) and stylistic inconsistencies that detract from clarity and professionalism. The abstract should be carefully edited for language precision, coherence, and alignment with standard structured abstract conventions.

**Introduction**

 Improve the introduction by expanding. Add the following references in the introduction to improve the background with recent findings: Ref 1, 2 and 3.

Is the work clearly and accurately presented and does it cite the current literature?

Yes

If applicable, is the statistical analysis and its interpretation appropriate?

Yes

Are all the source data underlying the results available to ensure full reproducibility?

Yes

Is the study design appropriate and is the work technically sound?

Yes

Are the conclusions drawn adequately supported by the results?

Yes

Are sufficient details of methods and analysis provided to allow replication by others?

Partly

Reviewer Expertise:

Haematology, Immunology, Public Health, Oncology

I confirm that I have read this submission and believe that I have an appropriate level of expertise to confirm that it is of an acceptable scientific standard, however I have significant reservations, as outlined above.

## References

[1] : Hematological factors in women with Asherman syndrome and primary infertility: An overview. *Medicine* .2025;104(43) : 10.1097/MD.0000000000045562 10.1097/MD.0000000000045562 PMC1255831141137354

[2] : Infertility: Prevalence and consequences. *Int J Curr Res Chem Pharm Sci* .2023;

[3] : Antioxidants as Potential Therapeutics for Improving Female Fertility: A Narrative Review. *Int J Curr Res Chem Pharm Sci* .2024;

# Response

**Published:** 2026-03-08

**Authors:** Deema AL Shawan

**Affiliations:** Imam Abdulrahman Bin faisal University, Saudi Arabia

Dear reviewer,

 Thank you for your valuable input.

 Blew, please find  point-by-point responses to your comments regarding our abstract and introduction section accordingly.

**Comment: “ Background**

 The background section is overly broad and descriptive, lacking a clear articulation of the specific knowledge gap this study addresses. While infertility prevalence is mentioned globally and nationally, the abstract does not sufficiently justify *why* patients’ perceptions and barriers in Khobar and Dammam warrant focused investigation, nor how this study advances existing literature. The authors should explicitly state the unmet research gap and the study’s unique contribution.”

**Response:**

 This was done as well as I could without exceeding the word limit of the abstract, which is three hundred words.

**Comment: “ Objectives Require Greater Clarity and Analytical Focus**

 The study aims are stated in a generalized manner and conflate perceptions, service evaluation, and barriers without clearly distinguishing primary and secondary objectives. The abstract should clearly specify whether the central focus is patient perception, service accessibility, systemic barriers, or policy implications, and how these components are analytically linked.”

**Response:**

 There are two objectives (aims) to the study, both of which have been edited to make them more concise and briefly explained in the background section of the abstract.

**Comment “Methodological Description Lacks Rigor and Transparency**

 The methods section is insufficiently detailed for a qualitative study. The term “qualitative, hybrid thematic analysis” is vague and not adequately explained. The abstract should briefly clarify what is meant by “hybrid” (e.g., inductive–deductive), the data collection method (e.g., interviews, focus groups), and the rationale for including three distinct participant groups. Additionally, justification for the small sample size (n=15) and its adequacy for thematic saturation is absent.”

**Response:**

 The recommended information has been added to the methods section, but was kept brief due to the concise nature of the abstract and further elaborated in the manuscript.

**Comment “ Results Section Is Incomplete and Conceptually Inconsistent**

 There is an inconsistency in the reporting of themes: the abstract states that *four* themes were identified but lists only *three*. This is a major structural flaw. Furthermore, the results are presented at a superficial level, largely restating common assumptions (e.g., financial burden, societal influence) without highlighting novel, context-specific insights. The authors should succinctly articulate the most salient and distinctive findings emerging from the data.”

**Response:**

 Thank you for highlighting the error it was corrected and further elaboration were added to the results in the abstract.

**“Conclusions Are Overgeneralized and Not Fully Grounded in the Results**

 The conclusions section introduces policy recommendations (e.g., case management, insurance coverage) that are not clearly tied to the reported findings within the abstract. The authors should ensure that conclusions logically flow from the stated results and avoid introducing new concepts not previously mentioned. The implications should be framed cautiously, reflecting the qualitative nature and limited sample size of the study.”

**Response:**

 In accordance with this recommendation, the conclusion section in the abstract  was edited accordingly.

**Comment:** “ **Language, Structure, and Technical Accuracy Require Improvement**

 The abstract contains grammatical errors (e.g., “his study” instead of “This study”) and stylistic inconsistencies that detract from clarity and professionalism. The abstract should be carefully edited for language precision, coherence, and alignment with standard structured abstract conventions. **“**

**Response:**

 Thank you for your feedback.

  All grammatical errors have been corrected.

**Comment” Introduction**

 Improve the introduction by expanding. Add the following references in the introduction to improve the background with recent findings: Ref 1, 2 and 3.”

**Response:**

 I thoroughly read the three references and found them very insightful. I have included Reference 2 since it is most relevant to our study.

# Response

**Published:** 2026-05-19

**Authors:** Deema AL Shawan

**Affiliations:** Imam Abdulrahman Bin faisal University, Saudi Arabia

Thank you for your valuable feedback, please refer to our response in our previous reply.

 Kindly let us know if you have further comments, we are still looking forward to your response.

# Reviewer report: Reviewer response for version 1

**DOI:** 10.5256/f1000research.185167.r438620

**Published:** 2025-12-16

**Authors:** Asmaa Alyaemni

**Affiliations:** 1 King Saud University, Riyadh, Riyadh Province, Saudi Arabia

1.the research question clearly stated and suitable for a qualitative approach

 2. Sampling strategy was not specified.

 3. The data collection is rigour yet interview guide were not included.

 3. the description of the analysis approach should be more clear (thematic analysis, framework analysis, grounded theory coding) and Step-by-step explanation of coding ,e.g. use of multiple coders or peer checking and also evidence of data saturation needs some illustration 

 4.Trustworthiness (Rigor Criteria); qualitative studies use trustworthiness Criterion. Teh author might want to look for:

 Credibility: Member checking, prolonged engagement

 Dependability: Audit trail, clear methodology

 Confirmability: Reflexivity, researcher positionality

 Transferability: Thick description, context detail

 Overall, the paper is very strong and can be indexed.

Is the work clearly and accurately presented and does it cite the current literature?

Yes

If applicable, is the statistical analysis and its interpretation appropriate?

Yes

Are all the source data underlying the results available to ensure full reproducibility?

No source data required

Is the study design appropriate and is the work technically sound?

Yes

Are the conclusions drawn adequately supported by the results?

Yes

Are sufficient details of methods and analysis provided to allow replication by others?

Yes

Reviewer Expertise:

Heath care administrations research, qualitative studies

I confirm that I have read this submission and believe that I have an appropriate level of expertise to confirm that it is of an acceptable scientific standard.
